# Prazosin use in a patient with rare *Neurobeachin* gene deletion shows improvement in paranoid behavior: a case report

**DOI:** 10.1186/s13256-021-03209-2

**Published:** 2021-12-24

**Authors:** Christina Y. Cantwell, Jamie Fortman, Alexis Seegan

**Affiliations:** UC Irvine Medical Center, Department of Psychiatry and Human Behavior, 101 The City Drive South, Orange, CA 92868 USA

**Keywords:** Prazosin, Neurobeachin, Autism spectrum disorder, Paranoia, Fear response, Case report

## Abstract

**Background:**

Disruption of the *Neurobeachin* gene is a rare genetic mutation that has been implicated in the development of autism and enhanced long-term potentiation of the hippocampal CA1 region, causing a heightened conditioned fear response and impaired fear extinction. Prazosin, an alpha-1 receptor antagonist, has been used in patients with posttraumatic stress disorder to mitigate the increased alpha-1 activity involved in fear and startle responses. Here we report a case of a patient with a rare *Neurobeachin* gene deletion, who demonstrated marked and sustained improvement in paranoid behavior within days of prazosin initiation.

**Case presentation:**

The patient is a 27-year-old White male with autism spectrum disorder, obsessive–compulsive disorder, and schizophrenia, with a chromosome 13q12 deletion including deletion of the *Neurobeachin* gene, who presented to the emergency department due to worsening functional status and profound weight loss as a result of only eating prepackaged foods. He had not showered or changed clothes in several months prior to presentation. He was hospitalized in the inpatient psychiatric unit for 2 months before prazosin was initiated. During that time, he demonstrated paranoia as evidenced by heightened sensitivity to doors opening, guarded interactions, and limited communication with providers and other patients. He also exhibited poor grooming habits, with aversion to showering, shaving, and changing clothes. Since initiating prazosin, he has demonstrated a brighter affect, initiates and maintains conversations, showers and changes clothes on a regular basis, and eats a variety of foods. At the time of this report, the patient was discharged to live in an apartment with a caregiver after a 7-month inpatient hospitalization.

**Conclusions:**

Low-dose prazosin shows rapid and sustained improvement in paranoid behavior in a patient with a rare *Neurobeachin* gene deletion. Prazosin has a relatively favorable side effect profile with once-daily dosing and low cost. Prazosin may provide clinical improvement in patients with *Neurobeachin* gene deletions due to its theoretical attenuation in fear response through alpha-1 antagonism.

## Background

Neurobeachin (NBEA) is a neuron-specific protein with high affinity for the type II regulatory subunit of protein kinase A that is involved with vesicle trafficking and signal transduction [1]. *Nbea* has been described as a candidate gene for autism and appears to play a role in neurodevelopment, with high expression in the central nervous system during the prenatal period, followed by a rapid decline by nearly half during the first 25 days of life [2, 3]. Castermans *et al.* [3] previously reported a case of *neurobeachin* gene disruption by translocation in a patient with idiopathic autism.

The *Nbea* deletion may play a role in the development of autism [4–6]. One mouse model of *Nbea* knockout showed evidence to support the gene’s role in the excitatory–inhibitory imbalance model of autism [4]. Other phenotypes associated with *neurobeachin* gene deletion include epilepsy, delayed speech onset, and delayed gross motor skills [5].

*Nbea* gene deletions have also been implicated in neurodevelopmental disorder with or without early onset generalized epilepsy (NEDEGE) [7]. Patients with NEDEGE may demonstrate intellectual disability, learning difficulties, behavioral dysregulation, autistic features, and epilepsy. The patient we describe in this report had some of these features and those described below, though did not have a history of seizures.

Nuytens *et al.* [8] and Lee *et al.* [9] report *Nbea* haploinsufficiency can cause increased long-term potentiation in the CA1 region of the hippocampus, leading to an enhanced conditioned fear response and impaired fear extinction. Prazosin is an alpha-1 adrenergic receptor antagonist that has been useful in treating patients with posttraumatic stress disorder (PTSD), likely due to the increased alpha-1 adrenergic activity seen in heightened startle responses [10]. Ketenci *et al.* [11] describe the use of prazosin in PTSD models to successfully alleviate stress responses.

To our knowledge, there have been no other cases of prazosin use to mitigate the increased fear response and diminished extinction in patients with an *Nbea* deletion, in part due to the low frequency of the genomic abnormality in the general population, as well as barriers to accessing microarrays on a large scale to determine an accurate incidence of *Nbea* disruptions.

Here, we describe a case of treating a patient with a known *Nbea* gene deletion confirmed by microarray, with low-dose prazosin as a therapeutic method to improve his paranoid and hypervigilant behavior. At the time of this report, he has been treated with 2 mg prazosin for 5 months, with marked improvement in stress response as evidenced by improvement in interpersonal communication and diminished sensitivity to surroundings.

## Case presentation

The patient is a 27-year-old White male with autism spectrum disorder (ASD), obsessive–compulsive disorder (OCD), and chromosome 13q12 deletion who initially presented to the emergency department with his parents for worsening functional status. He is an only child with no family history of autism, born at full term without complications.

Prior to hospitalization, he was on escitalopram 20 mg, lamotrigine 200 mg, and ziprasidone 40 mg, which his parents noted became ineffective over the previous 5 years. Regarding prior diagnoses, the patient was diagnosed with autism spectrum disorder in early childhood after neuropsychiatric testing and held this diagnosis when he was first evaluated by our psychiatrists in his 20s. Obsessive–compulsive disorder was also diagnosed prior to hospitalization based on his rigid routine and only eating prepacked foods. The diagnosis of schizophrenia was made in the months prior to hospitalization as the patient developed paranoia and isolation over the previous 6 years, with a significant decline in executive, social, and occupational function over the last year. In high school, he was noted to be social and cheerful, and he gradually became more isolated and withdrawn, stopped using technological devices such as a TV or phone, and had a 70 lb weight loss over 1 year as a result of eating only small portions of prepackaged food from a particular store and skipping dinner. He had not showered or changed clothes in several months. When he arrived at the psychiatric unit, he displayed paranoia, poverty of speech, perseveration, poor hygiene, and regimented routine.

While the patient exhibited a decline in oral intake with eating selective meals, we doubt medication nonadherence during the period preceding his hospitalization for several reasons. The patient is conserved by his parents who facilitate his appointments with his psychiatrist and keep track of his medication refills, which ran out at the expected time. The patient also followed a strict regimen with medication administration at specific times of the day. Additionally on intake labs, the lamotrigine level was within therapeutic range at 6.3 mcg/mL, with a negative urine drug screen.

While on the ward, he was hypervigilant, most notably when engaging in conversation, as he would look over his shoulder when doors opened or others walked by him. He displayed poverty of thought and often responded with “I don’t know” or “I’m alright” in an echolalic manner. Focus was placed on changing the medications for psychosis management given the patient’s worsening functional status. Ziprasidone was ineffective prior to admission, likely as a result of poor oral intake, and was transitioned to risperidone. Additionally, risperidone’s potential for long-acting injectable via paliperidone would have lowered his oral medication burden. Risperidone was trialed for 3 weeks, titrated to 4 mg twice a day (BID), and also deemed to be ineffective as the patient’s paranoia and functional status remained unchanged. As risperidone was decreased, olanzapine was started for psychosis; it was titrated to 20 mg BID. During this time, old records, including a CYP450 genotyping panel, were brought in by the family, and showed that the patient was an ultrarapid metabolizer of CYP1A2, the primary metabolic pathway for olanzapine, so aripiprazole was cross-titrated after 2 weeks of olanzapine trial. In total, he was on olanzapine for 4 weeks without significant clinical improvement. Aripiprazole was increased to 20 mg daily. Mild improvement in paranoia was noted on aripiprazole, as evidenced by his willingness to allow the treatment team to assess a chronic leg wound that he had previously kept hidden, although his mental status examination was otherwise unchanged. His home medication of escitalopram was continued and ultimately increased for treatment of OCD. Lamotrigine was also continued for mood stabilization throughout his hospital course.

With his known history of a chromosomal deletion, a microarray was performed. Results were significant for a pathogenic 9.5 Mb 13q13.2q14.11 deletion, which included the *Nbea* gene. Despite having tried multiple antipsychotics and increasing escitalopram over the course of his hospitalization, he continued to exhibit features of OCD and avoidance of certain textures (for example, water from shower, soft foods and vegetables), and continued to display heightened sensitivity and paranoia to his surroundings (for example, looking over shoulder to passersby or when doors open). These fear and paranoia symptoms seemed to be refractory despite trials of adequate doses of multiple antipsychotics for the first 2 months of his hospitalization, leading the treatment team to consider if the gene deletion was a possible explanation. Based on the published data on the effects of the *Nbea* deletion as outlined previously, in combination with the proposed mechanism of action of prazosin, the alpha-1 antagonist was started at a low dose, with noticeable improvement in paranoia within 2 days of initiation. The marked improvement in a short period provided a possible link to the exaggerated fear response seen in *Nbea* haploinsufficiency models. He became more conversational, disclosing more details about his day and mood, and engaged in more regular hygiene (showering, shaving, changing clothes). He was also noted to begin engaging with other peers on the ward, initiating conversations with others in a manner he had not previously displayed.

Prazosin was chosen as a potential treatment regimen due to its well-studied effects on alpha-1 adrenergic antagonism. While this patient did not have a triggering stressful event to lead to a diagnosis of posttraumatic stress disorder, his rare gene deletion may have been involved in diminished fear response extinction, creating a PTSD-like effect. A dose of 1 mg was initiated with no adverse effects including orthostatic hypotension, and the final dose of 2 mg was started 3 days later. Prazosin was well tolerated and continues to be a part of the patient’s regular medication regimen.

After a 7-month inpatient hospitalization, he was discharged to live in an apartment with a 24/7 caregiver. The lengthy hospitalization was due to discharge planning as his conservators were hesitant to discharge to independent living, but he did not qualify for an acute level of care and his medication regimen remained stable. At the time of this case report, the patient has been seen in the outpatient setting twice, and noted to be stable in his mental health with escitalopram 40 mg, lamotrigine 200 mg, and transitioned to aripiprazole long-acting injectable 400 mg monthly. He self-adjusted prazosin to 4 mg nightly and was evaluated in the interim without adverse effects, so he is continued at 4 mg as the current dose. The patient has been on prazosin nightly for 7 months with near-resolution of paranoid behavior. Now he initiates and engages in conversations, dances with others, showers and changes clothes without prompting, is less suspicious of his surroundings, and eats a wider variety of foods, resulting in appropriate weight gain from 65 kg to 72 kg since prazosin initiation. His affect has also improved from flat to reactive.

## Discussion and conclusions

The *Nbea* gene deletion is a rare chromosomal aberrancy, and its effects have been primarily studied in mice. In this case, the patient had a known chromosomal deletion, prompting the use of microarray to determine any potential detrimental gene disruptions, leading to the discovery of this patient’s *Nbea* deletion. In studies with decreased *Nbea* expression, fear acquisition and memory are seemingly not affected, while fear extinction is impaired, resulting in perpetuation of the fear response [9]. While it is not common practice to obtain microarrays for all patients with ASD, paranoia, or schizophrenia, improvements in cost and accessibility may facilitate the use of genetic analysis in patients who exhibit severe clinical features that are refractory to standard medication regimens. Smith *et al.* [12] report the case of a patient with sporadic autism and language deficit disorder who was also found to have a similar 13q12–13q13 deletion; however, the low prevalence of the *Nbea* deletion remains a limitation of our study as its effects have not been well studied in humans, and to our knowledge, there has been no human model for prazosin use in patients with an *Nbea* deletion.

Prazosin has been used as an antihypertensive and in PTSD treatment because of its known antagonism of alpha-1-adrenergic receptors and its favorable side effect profile. The medication can be dosed once daily as low as 1 mg, and its cost is relatively affordable. Lucas *et al.* [13] recently reported enhanced fear extinction in mice models when prazosin is used during the time period of fear conditioning, which may provide further improvement in mitigating the formation of new fear response with chronic use.

Given the rare nature of the patient’s *Nbea* gene deletion, the correlation with improvement in paranoid behavior with prazosin is speculative at this point, and further controlled studies are needed, although difficult to conduct in human subjects. Prazosin may be helpful as adjunct therapy to improve functioning and decrease fear, as the *Nbea* deletion may be contributing to a more severe clinical picture in a patient with underlying schizophrenia, ASD, and OCD.

In this patient, noticeable rapid improvement was seen at a low dose of 1–2 mg prazosin within days of treatment initiation. This case report highlights the potential for prazosin in targeting the enhanced fear conditioning that is seen in *Nbea* deletions through its direct pharmacologic properties. In this case, the patient was able to attend to his activities of daily living more appropriately, including improved hygiene, eating, and interpersonal communication, ultimately resulting in a discharge to an apartment after 7 months of inpatient psychiatric hospitalization.

## Data Availability

Not applicable.

## Abbreviations

NBEA: Neurobeachin
ASD: Autism spectrum disorder
PTSD: Posttraumatic stress disorder
OCD: Obsessive–compulsive disorder
NEDEGE: Neurodevelopmental disorder with or without early onset generalized epilepsy
OMIM: Online Mendelian Inheritance in Man
